# Isoniazid Preventive Therapy and Pregnancy Outcomes in Women Living With Human Immunodeficiency Virus in the Tshepiso Cohort

**DOI:** 10.1093/cid/ciz1024

**Published:** 2019-10-21

**Authors:** Nicole Salazar-Austin, Silvia Cohn, Sanjay Lala, Ziyaad Waja, Kelly E Dooley, Christopher J Hoffmann, Richard E Chaisson, Neil Martinson

**Affiliations:** 1 Center for Tuberculosis Research, Johns Hopkins University School of Medicine, Baltimore, Maryland, USA; 2 Department of Paediatrics, Chris Hani Baragwanath Hospital and University of the Witwatersrand, Soweto, South Africa; 3 Perinatal HIV Research Unit, University of the Witwatersrand, Soweto, South Africa

**Keywords:** tuberculosis, pregnancy, isoniazid preventive therapy (IPT), pregnancy outcomes

## Abstract

**Background:**

Both pregnancy and human immunodeficiency virus (HIV) increase the risk of tuberculosis disease, which results in poor maternal, pregnancy, and infant outcomes. Isoniazid preventive therapy (IPT) reduces mortality among individuals living with HIV in high-burden settings but has recently been associated with adverse pregnancy outcomes when initiated during pregnancy.

**Methods:**

In this secondary analysis, we used multivariable logistic regression to evaluate the association between IPT exposure and adverse pregnancy outcomes (fetal demise, prematurity, low birth weight, congenital anomaly) in pregnant women living with HIV enrolled as controls in the Tshepiso study, a prospective observational cohort of pregnant women living with HIV with and without tuberculosis disease in Soweto, South Africa, from 2011–2014.

**Results:**

There were 151 women enrolled with known pregnancy outcomes; 69 (46%) reported IPT initiation during pregnancy. Of the 69 IPT-exposed women, 11 (16%) had an adverse pregnancy outcome compared with 23 (28%) IPT-unexposed women. The adjusted odds of having an adverse pregnancy outcome was 2.5 (95% confidence interval, 1.0–6.5; P = .048) times higher in IPT-unexposed women compared with IPT-exposed women after controlling for maternal age, CD4 count, viral load, antiretroviral regimen, body mass index, and anemia.

**Conclusions:**

IPT exposure during pregnancy was not negatively associated with pregnancy outcomes after controlling for demographic, clinical, and HIV-related factors. These results provide some reassurance that IPT can be safely used in the second or third trimester of pregnancy. Additional research is needed to evaluate the safety of IPT and new short-course tuberculosis preventive therapies during pregnancy.

## Teaser

In this secondary analysis of pregnant women living human immunodeficiency virus (HIV) with and without isoniazid exposure for tuberculosis prevention during pregnancy, we did not observe a negative association between isoniazid preventive therapy use and pregnancy outcomes after controlling for demographic, clinical, and HIV-related confounding factors.

Tuberculosis (TB) remains the leading infectious cause of death worldwide, killing 476 000 women in 2017 [1]. Both pregnancy and human immunodeficiency virus (HIV) increase a woman’s risk of TB disease [2, 3]. In sub-Saharan Africa, the World Health Organization (WHO) estimates a 10-fold higher prevalence of TB disease among pregnant women living with HIV compared with pregnant women not living with HIV [4]. Maternal TB disease has devastating consequences for both mothers and infants including increased maternal and infant mortality, adverse pregnancy outcomes including prematurity and low birth weight, infant TB, and perinatal HIV transmission [5–15].

Isoniazid preventive therapy (IPT) in people living with HIV (PLHIV) in high TB burden settings provides a durable benefit on preventing both TB and mortality, independent of and strengthened by coadministration of combination antiretroviral therapy (cART) [16, 17]. However, the safety of IPT in pregnancy is not well established, and concerns remain for increased risk of maternal hepatotoxicity [18, 19]. Nevertheless, IPT has been recommended for pregnant women living with HIV in high-burden settings since 1993 [20]. TB APPRISE, a phase 4 randomized, controlled trial evaluating the safety and timing of IPT initiation among pregnant women living with HIV in high TB burden settings, reported no difference in maternal hepatotoxicity but, unexpectedly, found a higher risk of adverse pregnancy outcomes (fetal demise, prematurity, low birth weight, and congenital anomaly) among mothers who received IPT during pregnancy than in the postpartum period [21]. Two additional observational substudies of TB preventive therapy trials assessing the use of daily and weekly isoniazid during pregnancy or at conception found no association between isoniazid use and adverse pregnancy outcomes [22, 23].

Defining the association between isoniazid and adverse pregnancy outcomes is crucial given the large number of pregnant women living with HIV in low-resource settings at extreme risk of TB disease and its maternal and infant complications and the well-established survival benefit of IPT in this setting. Given the unexpected association between IPT and adverse pregnancy outcomes found in TB APPRISE and the conflicting observational data, we aimed to add to the body of knowledge on the safety of IPT in pregnancy. Here, we examined the association between IPT and pregnancy outcomes in a secondary analysis of a well-characterized prospective cohort of pregnant women living with HIV in Soweto, South Africa, from 2011–2014.

## METHODS

### Study Design

Tshepiso was a prospective matched cohort study of pregnant women living with HIV with and without TB disease. Pregnant women were recruited from 10 prenatal clinics in Soweto, South Africa, from January 2011 through January 2014 [5]. The study enrolled pregnant women living with HIV aged ≥18 years and at least >13 weeks’ gestation. For each woman with TB disease, 2 pregnant women living with HIV without TB disease were enrolled as controls and matched by age (±5 years), gestational age (±2 weeks), date of their first prenatal visit (±8 weeks), and, to avoid bias by indication for place of delivery, their planned delivery clinic. All women had a sputum sample taken for liquid mycobacterial culture (BACTEC MGIT 960 System) to confirm TB disease in women with TB and to confirm absence of pulmonary TB in controls. This study was noninterventional; all women and children received care at public sector clinics in Soweto according to South African ART and TB guidelines. The Tshepiso study doctor offered IPT to all study participants without TB disease who had not been initiated on IPT at public sector clinics. Follow-up and isoniazid refills were provided to all participants at public sector clinics. Here, we report the maternal, pregnancy, and infant outcomes among pregnant women living with HIV without TB disease who did or did not report isoniazid use for TB prevention during pregnancy.

### Prevention and Treatment Guidelines for HIV and TB

For the first 2 years of the study, South African prevention of mother-to-child transmission (PMTCT) guidelines recommended WHO’s option A, that is, efavirenz (EFV)-based antiretroviral therapy when CD4 count is ≤350 and zidovudine (AZT) monotherapy, with single-dose nevirapine at the initiation of labor, intrapartum AZT, and 1 postpartum dose of tenofovir and emtricitabine when CD4 count is >350 [24]. In March 2013, South Africa began to recommend option B with EFV-based antiretroviral therapy either as prophylaxis for the duration of pregnancy and breastfeeding or lifelong cART for those with CD4 count <350 or a stage 3 or 4 illness [25].

For the duration of the study, IPT was recommended for all eligible individuals living with HIV prior to or after initiating cART for a 6-month duration [26]. IPT eligibility was assessed by clinical history only. PLHIV not eligible for IPT included those with symptoms of TB disease, active liver disease, and/or active alcohol abuse. Notably, pregnancy was not a contraindication.

### Data Collection

Mother–infant pairs were enrolled during the second or third trimester and followed at 1 week, 6 weeks, 6 months, and 12 months postdelivery for maternal, pregnancy, and infant outcomes including TB incidence and mortality. All outcomes were self-reported and confirmed using clinic and hospital records and the infant’s road-to-health card. IPT exposure was assessed by self-report at each visit and confirmed using medical records, when available. Laboratory testing was performed by Clinical Laboratory Services, an accredited commercial research laboratory.

### Exposures and Outcomes

For the purpose of this study, isoniazid exposure was defined as self-reported use of isoniazid for TB prevention of any duration while pregnant. Measured pregnancy outcomes included spontaneous abortion (<28 weeks), stillbirth (≥28 weeks), prematurity (<37 weeks), birth weight, size for gestational age, and congenital anomaly. Long-term outcomes included incident maternal and infant TB disease and infant and maternal mortality. Gestational age was determined by the date of the last menstrual period. Maternal mortality was defined as maternal death within 42 days of delivery and infant mortality as infant death within the first year of life. Low birth weight and small size for gestational age were defined as birth weight <2.5 kg and <10th percentile, respectively [27]. Measured confounding variables included CD4 count at enrollment (cells/µL), viral load at enrollment or nearer to delivery (copies/mL), PMTCT regimen at delivery, hemoglobin and body mass index (BMI) at enrollment, and sociodemographic factors including maternal age at enrollment, race, education, and income.

For this analysis, we defined 3 composite outcomes. First, adverse pregnancy outcome included fetal demise (spontaneous abortion <28 weeks and stillbirth >28 weeks), low birth weight (<2.5 kg), prematurity (<37 weeks), and/or major congenital anomaly (according to the Centers of Disease Control and Prevention Metropolitan Atlanta Congenital Defects Program [MACDP] criteria [28]), as defined in other PMTCT and TB studies [21, 29]. Second, severe adverse pregnancy outcome included fetal demise, very low birth weight (<1.5 kg), very preterm delivery (<33 weeks), and/or major congenital anomaly (MACDP criteria [28]). Third, maternal/infant TB or death included maternal mortality, fetal demise, infant mortality, and/or maternal or infant TB within 1 year of delivery, as defined in other maternal TB studies [21].

### Statistical Analyses

Demographic and clinical characteristics of pregnant women were described using proportions for categorical variables and means with standard deviations (SDs) and medians with interquartile ranges (IQRs) for normally and nonnormally distributed continuous variables, respectively. We used Pearson χ^2^ tests or Fisher exact tests to compare categorical variables and Student *t* tests or Mann-Whitney *U* tests to compare continuous variables. Multivariable logistic regression models were used to describe the association between IPT exposure (exposure of interest) and adverse pregnancy outcomes (composite outcome). We adjusted for potential confounders identified a priori including maternal age, CD4 count, viral load, antiretroviral regimen, BMI, and hemoglobin. Collinearity was assessed using variance inflation factors.

Maternal age, CD4 count, viral load, hemoglobin, and BMI were assessed as both continuous and categorical variables. CD4 count and viral load categories were informed by clinically relevant thresholds used at the time the study was conducted [30–33]. Categories for maternal age, BMI, and anemia (hemoglobin) were based on published categories [30, 34–37], notably prior established BMI categories used BMI measured in the first trimester.

We performed a complete case analysis. Loss to follow-up was considered random and not due to isoniazid exposure. Data were analyzed using SAS statistical software (version 9.4) [38].

### Ethical Approval

The University of Witwatersrand Human Research Ethics Committee and the Johns Hopkins Institutional Review Board approved this study. Written informed consent for all participants was obtained prior to enrollment.

## RESULTS

**Figure 1.**
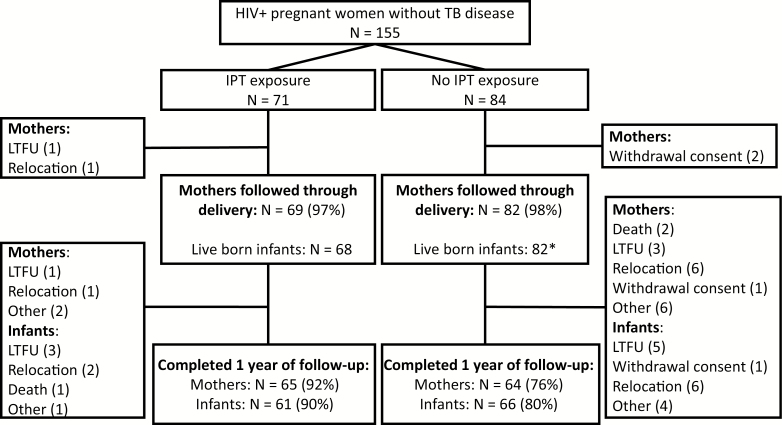
Participant flow diagram of pregnant women and infants born to women with and without isoniazid exposure during pregnancy. *Includes one set of twins. Abbreviations: HIV, human immunodeficiency virus; IPT, isoniazid preventive therapy; LTFU, loss to follow-up; TB, tuberculosis.

The study enrolled 155 women without TB disease (Figure 1). Of these, 71 (46%) reported use of IPT (IPT exposed) during pregnancy and 84 (54%) did not (IPT unexposed). Among IPT-exposed women, 1 woman relocated prior to delivery and 1 woman was lost to follow-up. Among IPT-unexposed women, 2 withdrew consent. Pregnancy outcomes were available for 69 (97%) and 82 (98%) women with and without IPT exposure, respectively. Long-term outcomes including TB disease and mortality, assessed at 1 year postdelivery, were available for 65 (92%) and 64 (76%) of IPT-exposed and IPT-unexposed women. Among IPT-exposed mothers, reasons for not completing follow-up were relocation (n = 1), loss to follow-up (n = 1), and other reasons (n = 2). Among IPT-unexposed mothers, reasons were relocation (n = 6), withdrawal of consent (n = 1), loss to follow-up (n = 3), death (n = 2), and other reasons (n = 6).

Among the 151 women who delivered, there was 1 set of twins and 2 stillbirths, resulting in 150 live births including 68 live births with IPT exposure in utero and 82 live births without IPT exposure in utero. A higher proportion of IPT-exposed infants (n = 61, 90%) were followed through infancy than IPT-unexposed infants (n = 66, 80%). Reasons for not completing a year of follow-up were similar across the 2 groups and included withdrawal of consent (n = 0 vs 1), relocation (n = 2 vs 6), loss to follow-up (n = 3 vs 5), death (n = 1 vs 0), and other reasons (n = 1 vs 4).

### Maternal Characteristics at Enrollment

Maternal characteristics at enrollment were similar in IPT-exposed and IPT-unexposed participants (Table 1). Both groups had a mean age of 29 years (SD, 4.6 and 3.8, respectively). Mean gestational age at enrollment was 29 weeks (SD, 5.8) in the IPT-exposed group and 31 weeks (SD, 5.3) in the IPT-unexposed group (*P* = .01). The IPT-exposed and IPT-unexposed groups had similar parity, number of prior miscarriages, median hemoglobin, median BMI, race, education, and employment.

**Table 1.** Characteristics of Maternal Pregnancy, Human Immunodeficiency Virus Disease, and Prior Tuberculosis Disease by Isoniazid Exposure

	IPT	No IPT	
Characteristic N = 151 Total	n = 69	n = 82	*P* Value
Sociodemographic characteristics at study enrollment			
Median maternal age at study enrollment (SD), y	29 (4.6)	29 (3.8)	.83
Number of participants contributing to this calculation	n = 69	n = 82	
Median gestational age at study enrollment (SD), wk	29 (5.8)	31 (5.3)	.01
	n = 69	n = 82	
Number of previous deliveries (%)			
0	2 (3)	7 (9)	.31
1	32 (46)	32 (39)	
2	23 (33)	27 (33)	
≥3	4 (6)	9 (11)	
Unknown	8 (12)	7 (9)	
Prior miscarriage, N (%)			.38
0	53 (77)	61 (74)	
≥1	8 (12)	14 (17)	
Unknown	8 (12)	7 (9)	
Race, N (%)			1.00
Black African	68 (99)	82 (100)	
Other	1 (1)	0 (0)	
Education, N (%)			
≤ 8th grade	6 (9)	8 (10)	.56
9th–12th grade	36 (52)	36 (44)	
Completed 12th grade	26 (38)	34 (41)	
Started or completed a tertiary degree	1 (1)	4 (5)	
Employment (prior 12 months), N (%)			
Currently employed	15 (22)	20 (24)	.72
Previously employed in the past year	19 (27)	18 (22)	
Unemployed in the last year	35 (51)	44 (54)	
Median hemoglobin at study enrollment (SD), g/dL	11.2 (1.3)	11.3 (1.3)	.70
Median body mass index at study enrollment (SD), kg/m^2^	27.8 (4.4)	28.4 (5.2)	.49
Characteristics of HIV infection at screening for study enrollment			
Median CD4 T-cell count at study enrollment (IQR), cells/μL	373 (275, 477)	364 (252, 464)	.36
CD4 categories at study enrollment, N (%), cells/mm^3^			
<100	0 (0)	3 (4%)	.32
100–349	26 (38)	36 (44)	
350–499	26 (38)	26 (32)	
≥500	16 (23)	16 (19)	
Unknown	1 (1)	1 (1)	
Prevention of mother-to-child transmission of human immunodeficiency virus regimen at delivery, N (%)			
Zidovudine monotherapy ± single-dose nevirapine	22 (32)	18 (22)	.45
Efavirenz + NRTI backbone	39 (56)	52 (63)	
Lopinavir/Ritonavir + NRTI backbone	2 (3)	3 (4)	
Nevirapine + NRTI backbone	4 (6)	8 (10)	
None	1 (2)	0 (0)	
Unknown	1 (2)	1 (1)	
Median duration of combination antiretroviral therapy prior to delivery (IQR), mo	4.7 (2.9, 6.7)	5.1 (3.5, 21.4)	.19
HIV viral load at or after study enrollment but prior to delivery, N (%), copies/mL			
<20 (undetectable)	27 (39)	45 (55)	.24
20–999	21 (30)	19 (23)	
1000–100 000	15 (22)	14 (17)	
>100 000	4 (6)	2 (3)	
Unknown	2 (3)	2 (2)	
Characteristics of prior TB disease and current isoniazid preventive therapy			
Prior episode of TB disease, N (%)			.25
0	62 (90)	68 (83)	
≥1	7 (10)	14 (17)	
Median gestational age at IPT initiation (SD), wk	25 (6.9)	-	-
IPT initiation, N(%)			
First trimester	2 (3)	-	-
Second trimester	33 (48)		
Third trimester	34 (49)		
Timing of IPT initiation, N(%)			
Prior to screening for study enrollment	33 (48)		
At screening for study enrollment	30 (43)		
After screening for study enrollment	6 (9)		
Median duration of isoniazid (SD), mo	3.4 (2.4)	-	-
Median duration of isoniazid while pregnant (SD), mo	2.4 (1.7)	-	-

Abbreviations: HIV, human immunodeficiency virus; IPT, isoniazid preventive therapy; IQR, interquartile range; NRTI, nonnucleoside transcriptase inhibitor; SD, standard deviation; TB, tuberculosis.

Baseline CD4 counts at enrollment were similar between the groups, 373 cells/µL (IQR, 275 to 477) among IPT-exposed participants and 364 cells/µL (IQR, 252 to 464) among IPT-unexposed participants. At delivery, similar proportions of participants received either AZT monotherapy with single-dose nevirapine at birth or cART. One participant refused both cART and AZT monotherapy for PMTCT but accepted IPT. The median duration of cART prior to delivery for IPT-exposed and IPT-unexposed women was 4.7 months (IQR, 2.9 to 6.8) and 5.4 months (IQR, 3.7 to 21.8), respectively, and a similar proportion of patients were virally suppressed. A similar proportion of IPT-exposed (10%) and IPT-unexposed (17%) women had at least 1 previous episode of TB (*P* = .25).

### IPT Exposure

Participants reported initiating IPT at a mean gestational age of 25 weeks (SD, 6.9; Table 1), with nearly half initiating IPT in the second (48%) vs third (49%) trimesters. No participants reported taking IPT at the estimated date of conception. Almost half of IPT initiations (48%) occurred at public sector clinics prior to study enrollment, 43% occurred at screening for study enrollment, and 9% occurred more than 1 month after study enrollment. The median reported duration of IPT during pregnancy was 2 months (IQR, 1 to 4). IPT was discontinued at a median of 5 days prior to delivery (IQR, −44 to 43).

### Pregnancy, Maternal, and Infant Outcomes by IPT Exposure

Of 69 pregnancies among IPT-exposed women, there was 1 stillbirth and 68 live births (Table 2). Of 82 pregnancies among IPT-unexposed women, there was 1 stillbirth and 1 set of twins, resulting in 82 live births. The median gestational age at birth was 39 weeks in both IPT-exposed (IQR, 38 to 40) and IPT-unexposed (IQR, 37 to 40) infants. There were 7 (10%) preterm deliveries among IPT-exposed pregnancies and 18 (22%) preterm deliveries among IPT-unexposed pregnancies (*P* = .06). There was no statistically significant difference in the proportion of infants with low birth weight (9% vs 12%; *P* = .60) or being small size for gestational age (12% vs 17%; *P* = .49) between IPT-exposed and IPT-unexposed groups, respectively. There were 3 congenital anomalies including 2 infants with extra digits and 1 infant with amelia of the right arm, representing 2% of live births in both groups (*P* = 1.0). No infants were diagnosed with TB in either group, and there was 1 infant death during the neonatal period in the IPT-exposed group. There were 2 maternal deaths during the year of follow-up including 1 death at less than 42 days and 1 death at 10 months, both in the IPT-unexposed group. One IPT-unexposed mother developed TB in the first year postdelivery.

**Table 2.** Pregnancy, Maternal, and Infant Outcomes by Isoniazid Exposure

	IPT	No IPT	
All Gestations	N = 69	N = 83	*P* Value
Fetal demise,^a^ N (%)	1 (1)	1 (1)	1.00
Live births only	N = 68	N = 82	
Median gestational age at birth (interquartile range), wk	39 (38,40)	39 (37,40)	.82
Preterm, N (%), wk	7 (10)	18 (22)	.06
34–36	5	17	
28–33	2	1	.18
<28	0	0	
Low birth weight (<2.5 kg), N (%)	6 (9)	10 (12)	.60
Very low birth weight (<1.5 kg), N (%)	2 (3)	0 (0)	.20
Small for gestational age (<10th %), N (%)	8 (12)	14 (17)	.49
Congenital anomaly, N (%)	1 (2)	2 (2)	1.00
Infant deaths, N (%)	1^b^ (2)	0 (0)	.45
Infant TB disease, N (%)	0 (0)	0 (0)	
Infant hospitalization, N (%)			
1	8 (12)	7 (9)	.61
≥2	2 (3)	1 (1)	
Maternal events	N = 69	N = 82	
Maternal mortality (<42 Days), N(%)	0 (0)	1 (1)	1.00
Maternal TB disease, N(%)	0 (0)	1 (1)	1.0
Maternal hospitalization, N(%)			
During pregnancy	9 (13)	11 (13)	.99
At delivery	0 (0)	2 (2.4)	.50
Postpartum	1 (1.5)	2 (2.4)	1.00
Composite outcomes	N = 69	N = 83	
Adverse pregnancy outcome,^c^ N(%)	11 (16)	23 (28)	.08
Severe adverse pregnancy outcome,^d^ N(%)	4 (6)	4 (5)	1.00
Maternal/Fetal/Infant death or TB, N(%)	2 (3)	3 (4)	1.00

Abbreviations: IPT, isoniazid preventive therapy; TB, tuberculosis.

^a^Fetal demise is spontaneous abortion and stillbirth.

^b^This death occurred in the neonatal period.

^c^Composite outcome includes fetal demise, low birth weight (<2.5 kg), prematurity (<37 weeks), and congenital anomaly.

^d^Composite outcome includes fetal demise (stillborn + spontaneous abortion), very low birth weight (<1.5 kg), very preterm delivery (<33 weeks), or major congenital anomaly.

Similar proportions of mothers were hospitalized during pregnancy, at delivery, and in the postpartum period in the IPT-exposed and IPT-unexposed groups. Medical record review did not suggest IPT discontinuations or hospitalizations were due to adverse events including isoniazid-associated hepatotoxicity.

**Figure 2.**
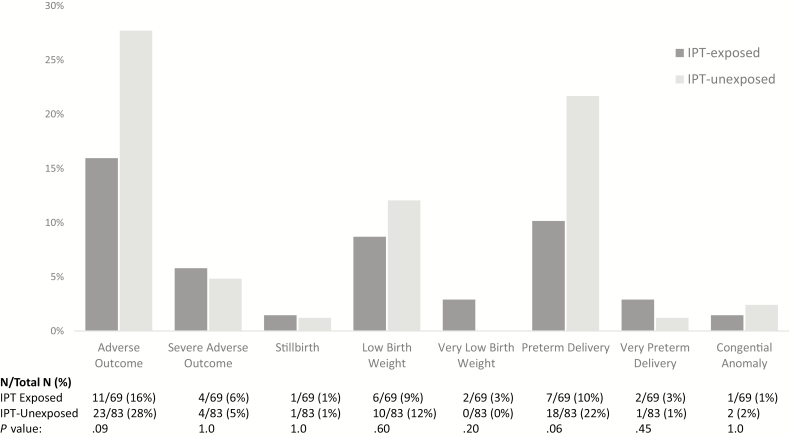
Pregnancy outcomes by isoniazid exposure. Composite and individual adverse pregnancy outcomes by isoniazid preventive therapy exposure. Adverse pregnancy outcome was a composite of fetal demise (spontaneous abortion and stillbirth), low birth weight (<2.5 kg), preterm delivery (<37 weeks), and major congenital anomaly. Severe adverse pregnancy outcome was a composite of fetal demise, very low birth weight (<1.5 kg), very preterm delivery (<32 weeks), and major congenital anomaly. Abbreviation: IPT, isoniazid preventive therapy.

There were half as many adverse pregnancy outcomes among IPT-exposed maternal–infant pairs (n = 11, 16%) than among IPT-unexposed maternal–infant pairs (n = 23, 28%; *P* = .08; Figure 2). Similar proportions of mother–infant pairs had severe adverse pregnancy outcomes in the IPT-exposed (n = 4, 6%) and IPT-unexposed (n = 4, 5%) groups (*P* = 1.0). There was no difference in maternal, fetal, or infant death or TB disease between those IPT-exposed and IPT-unexposed maternal–infant pairs (3% vs 4%; *P* = 1.0; Table 2).

In adjusted logistic regression analysis, IPT-unexposed women had 2.5 (95% confidence interval [CI], 1.0 to 6.5; *P* = .048) times the odds of having an adverse pregnancy outcome compared with IPT-exposed women (Table 3) after controlling for CD4 count, PMTCT regimen, viral load, maternal age, BMI, and anemia. This association did not significantly change in models with continuous vs categorical predictors (data not shown). In this model, women with advanced maternal age (≥35 years) have an adjusted odds ratio of having an adverse pregnancy outcome that is 0.6 (95% CI, .1 to 2.4; *P* = .50) times that of women aged <35 years, after controlling for IPT use, CD4 count, PMTCT regimen, viral load, BMI, and anemia. When age is modeled continuously, there is no difference in the adjusted odds of having an adverse pregnancy outcome (adjusted odds ratio, 0.9 to 95% CI, .8 to 1.0) with 1-year increments in age.

**Table 3.** Multivariable Logistic Regression for Having an Adverse Pregnancy Outcome

Risk factors N = 143	N (%)	Unadjusted OR	95% CI	Adjusted OR	95% CI
Isoniazid (tuberculosis prevention)					
Yes	69 (46)	1.0		1.0	
No	82 (54)	2.02	(.92 to 4.66)	2.51	(1.04 to 6.54)
Maternal age, y					
<35	133 (88)	1.0		1.0	
≥35	18 (12)	0.40	(.06 to 1.50)	0.58	(.08 to 2.41)
CD4 (per 50 cells/mm^3^ increments)	150 (99)	1.06	(.96 to 1.17)	1.09	(.98 to 1.22)
Viral load, copies/mL					
<1000	112 (74)	1.0		1.0	
≥1000	35 (23)	1.36	(.54 to 3.22)	1.82	(.56 to 5.80)
Unknown	4 (3)				
Prevention of mother-to-child transmission of human immunodeficiency virus regimen					
Combination antiretroviral therapy	108 (72)	1.0		1.0	
Zidovudine monotherapy/no therapy	41 (27)	1.09	(.46 to 2.49)	0.88	(.27 to 2.66)
Unknown	2 (1)				
Body mass index, kg/m^2^					
≥ 21.5	143(95)	1.0		1.0	
< 21.5	6 (4)	3.80	(.67 to 21.45)	3.35	(.57 to 19.73)
Unknown	2 (1)				
Anemia, g/dL					
Hemoglobin ≥8.5	145 (96)	1.0		1.0	
Hemoglobin <8.5	3 (2)	7.13	(.66 to 156.11)	11.97	(.99 to 286.19)
Unknown	3 (2)				

There were 143 of 152 participant pairs who had complete data and were included in the multivariable analysis.

Abbreviations: CI, confidence interval; OR, odds ratio.

## DISCUSSION

There were 2 principle findings in this prospective observational cohort study of pregnant women living with HIV. First, we found a protective association between IPT use and adverse pregnancy outcomes after controlling for potential demographic, clinical, and HIV-related confounding factors. Second, there were no clinically relevant episodes of maternal hepatotoxicity during pregnancy or postpartum among IPT-exposed women.

While IPT was found to have a protective association with pregnancy outcomes, the mechanism for this protection remains unclear given the lack of maternal and infant TB diagnoses or mortality among IPT-unexposed participants. Importantly, no negative association was found. These results are comparable to other observational data of TB preventive therapy in pregnant women living with HIV, including 2 substudies of clinical trials assessing either daily isoniazid or weekly isoniazid/rifapentine for TB prevention [22, 23]. Our results differ from the results from TB APPRISE, where an association between IPT use during pregnancy and adverse pregnancy outcomes was reported. In our study and in TB APPRISE, women were enrolled late during the second and third trimesters, resulting in similar durations of IPT during pregnancy. Gupta et al noted that this association may be a chance finding, given the large number of outcomes assessed, and warrants confirmation [21]. Our findings do not support their finding.

While no clinically relevant hepatotoxicity was observed, we did not routinely monitor liver enzymes, and transient elevations may not have been identified. Additionally, early discontinuation of IPT limited our assessment of postpartum hepatotoxicity.

Known clinical and demographic risk factors associated with adverse pregnancy outcomes in other observational studies and clinical trials of populations living with and without HIV were modeled here as confounding factors. Point estimates for these effects are imprecise given the limited sample size. In our model, advanced maternal age appeared protective when commonly used thresholds for age were used. When analyzed as a continuous variable, maternal age was not associated with adverse pregnancy outcomes. This may be due to the small proportion of participants who were aged ≥35 years (11%). As expected, elevated viral load was associated with adverse pregnancy outcomes, but low CD4 count was not. This was a relatively healthy cohort, with few participants presenting with advanced HIV disease or severe immunosuppression. Protease inhibitors have been associated with adverse pregnancy outcomes [29, 39]. Only 5 women in this analysis had a cART regimen that contained a protease inhibitor during pregnancy; 2 (40%) had premature infants. Given our small sample size, we were unable to analyze specific cART regimens in the logistic regression analysis. Whether mediated through HIV disease or other factors, low BMI and anemia have also been associated with adverse pregnancy outcomes in pregnant women living with HIV [34], as seen here.

This study has several limitations. First, this was an observational study and, though we found few significant differences between groups on measured baseline factors and controlled for several potential demographic, clinical, and HIV-related confounding factors in our analysis, we cannot exclude the possibility of selection bias. It is possible that healthier women were initiated on IPT, thereby overestimating its effect on pregnancy outcomes. Second, given our limited sample size, we were unable to control for all relevant factors associated with adverse pregnancy outcomes including the specific cART regimen, timing of cART initiation with respect to conception, prior prematurity, obstetrical risk factors, and drug use including tobacco and alcohol. Third, while IPT duration was short, most women reported continuing IPT through delivery, allowing for an accurate estimation of IPT’s effect on birth outcomes. These outcomes were measured in >97% of all participants, leading to confidence in the results. Finally, our generalizability may be limited as our study was partially conducted during implementation of PMTCT option A.

In conclusion, we did not find a negative association between IPT during pregnancy and pregnancy outcomes, after controlling for CD4 count, viral load, PMTCT regimen, maternal age, BMI, and anemia. These results may provide some reassurance that IPT can be safely used in the second or third trimester of pregnancy in high-burden settings. Given the high risk of TB disease in pregnant women living with HIV and the resulting poor maternal and infant outcomes, we need additional research to confirm the safety of isoniazid during pregnancy both alone and in combination with rifapentine in new short-course TB preventive therapy regimens.

## Notes

***Financial support.*** This work was supported by the National Institutes of Child Health and Human Development (grant R01HD064354 to R. E. C.) and the National Institutes of Health (P30A1094189, 4KL2TR001077-04, and K23HD096973).

***Potential conflicts of interest.*** R. E. C. reports that his spouse is a shareholder in Merck, and he reports consulting fees from Otsuka and Sanofi outside the submitted work. N. M. reports grants to his institution from Roche, Becton-Dickinson, and Pfizer outside the submitted work. All other authors report no potential conflicts. All authors have submitted the ICMJE Form for Disclosure of Potential Conflicts of Interest. Conflicts that the editors consider relevant to the content of the manuscript have been disclosed.
